# Mobile genetic elements and wastewater treatment: contaminants of emerging concern, climate change, and trophic transmission

**DOI:** 10.3389/fmicb.2025.1699325

**Published:** 2025-11-10

**Authors:** Aaron Bradshaw

**Affiliations:** Society-Environment Research Group, Institute of Geography, Friedrich-Alexander-Universität Erlangen-Nüremberg, Erlangen, Germany

**Keywords:** MGE, HGT, PFAS, microplastics, contaminants of emerging concern, WWTP, AMR

## Abstract

This minireview focuses on recent developments regarding mobile genetic elements (MGEs) and horizontal gene transfer (HGT) in wastewater treatment plants (WWTPs) and proximal environments. WWTPs are often discussed as hotspots and bioreactors for the evolution of MGEs and ARGs and their horizontal transfer. Firstly, the article reviews the effects of emerging contaminants on HGT and MGEs with a specific focus on microplastics and per- and polyfluoroalkyl substances (PFAS). Secondly, the review focuses on how extreme weather and climate change can overwhelm WWTPs, increase the input of diverse genetic elements, and alter the dynamics of HGT. Finally, the trophic connections between the WWTP microbiota and external ecosystems underscore the potential for wider transmission of MGEs. Here, the focus is on transfer of MGEs to larger organisms in the vicinity of WWTPs. In sum, the review focuses on emerging areas of research that refine our understanding of the WWTP environment as a hotspot for HGT and dissemination of MGEs with potentially deleterious implications for human and wider ecosystem health.

## 1 Introduction

Horizontal genetic transfer (HGT) refers to the sharing of genetic material between microorganisms (Soucy et al., 2015). HGT events are mediated via several distinct mechanisms including conjugation, transduction, and transformation, and via gene transfer agents including plasmids and phages. HGT is a key driver of microbial genomic plasticity and is central to microbes’ ability to adapt to changing and/or stressful environmental conditions (Arnold et al., 2022). Along with the activity of transposons and integron/integrases which drive recombination events within the genome, HGT is an important source of genetic innovation and novelty in microorganisms (Carscadden et al., 2023). Increasingly complex and reticulated genomic structures such as plasmids with multiple drug resistances (Mbanga et al., 2021) and large integron cassettes are often the end products of successive HGT events (Ghaly et al., 2021). The transfer of MGEs is catalyzed by anthropogenic pressures (Gillings, 2017; Groussin et al., 2021; Bradshaw, 2024a) and is particularly intense within wastewater treatment plant (WWTP) environments. Due to their unique function and position in relation anthropogenic activities, WWTPs and their surrounding environments can be seen, on the one hand, as a rescaled microcosm (or model) of the wider socio-environmental dynamics that characterize the Anthropocene (see Achmon et al., 2018). On the other hand, attention to WWTPs can unearth key dynamics of microbial evolution and interaction, particularly HGT, as it adapts to an era of unprecedented environmental change. These interrelated processes are two sides of the same coin that foreground the entangled complexity of the WWTP and its connections to wider environments.

**FIGURE 1:**
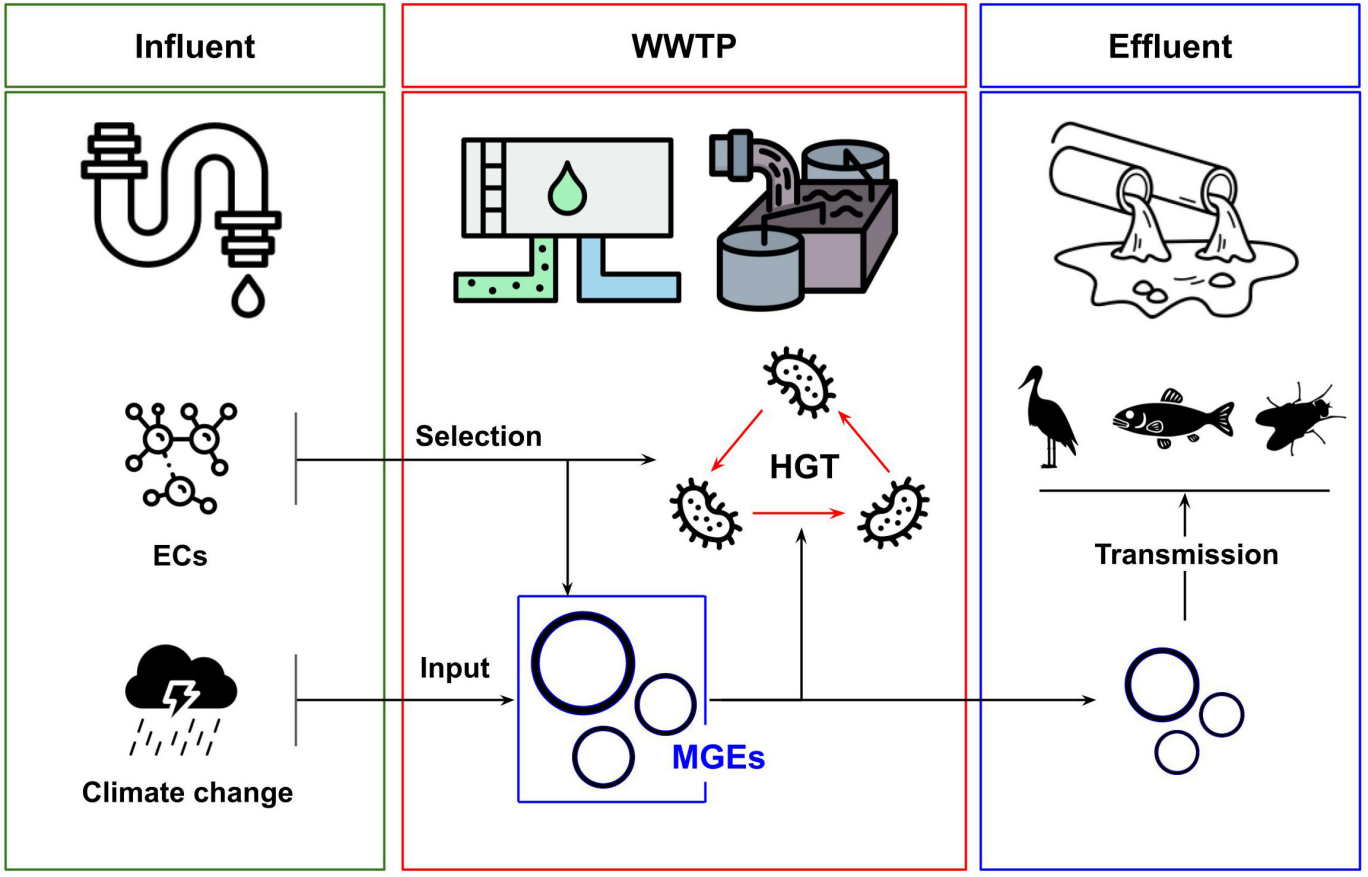
Overview of relationships between emerging contaminants (ECs), climate change and transmission of mobile genetic elements (MGEs) with wastewater treatment plant environments (WWTPs). ECs can select for increased rates of HGT and the abundance of MGEs. Strong weather events can influence the input of MGEs into WWTPs. The dissemination of MGEs in WWTP effluent can promote transmission to downstream ecologies, including larger organisms.

This minireview summarizes recent research focusing on HGT and MGEs in WWTPs. The aim is not to provide a systematic overview of the variables influencing HGT in these environments but to focus on areas that are currently emerging in the literature and which represent important areas for future study. Specifically, the focus is on the role of emerging contaminants, the impact of environmental volatility, and on identifying HGT events to the microbiomes of other ecosystems and species that live and feed in and around WWTPs. These three areas correspond to emerging hazards posed by WWTPs and their interfacing with an increasingly chemically diverse human society and anthropogenic climate change. They represent key sites for ongoing research and are schematically represented in Figure 1.

## 2 Wastewater treatment plants and horizontal gene transfer

Wastewater treatment plants (WWTPs) are unique ecological environments that collect and treat the effluent from human societies. Depending on the precise organization of the WWTP, this can include municipal/domestic wastewater and/or more specialized wastewater streams (hospital, agricultural, and/or industrial) as well as stormwater runoff. The primary method of wastewater treatment is the activated sludge process (AS) in which microbial aerobic digestion is enrolled to reduce the biological oxygen demand (BOD) of influent (McCabe and Eckenfelder, 1961) allowing the treated water to be returned to watersheds (oceans or rivers). The AS microbiome is a complex community that is characterized by a set of core microorganisms (Saunders et al., 2016), dominated by denitrifying bacteria (Yu and Zhang, 2012) and exhibits seasonal variation (Ju et al., 2014). In addition to the core functions of nutrient removal, the AS microbiome contains biodegradation genes targeted toward specific pollutants (e.g., Cai et al., 2013; Fang et al., 2013, 2018) although their function tends to be variable (Douziech et al., 2018).

The WWTP environment exposes a highly dense and diverse microbial community to various hard selection pressures including antibiotics, heavy metals, and disinfectants, along with emerging contaminants of concern (see below). This in turn promotes bacterial evolution and the sharing of genetic material (Brown et al., 2024; Fang et al., 2024). Because of the nature of these selective agents and their effects on bacterial communities, WWTPs are increasingly recognized as “hotspots” (Guo et al., 2017) that foster the emergence and evolution of antimicrobial resistance (AMR; see Bradshaw, 2024b). Firstly, the WWTP community can itself harbor ARGs (on MGEs), thereby acting as an AMR “reservoir” (Yoo et al., 2020; Yin et al., 2022; Zhang et al., 2022). Secondly, the release of MGEs from WWTPs can promote the transfer of ARGs to downstream ecosystems and environments (Sambaza and Naicker, 2023). These processes underscore the framing of WWTPs as risky environments that both concentrate and disperse various hazards.

Over the last century or so HGT has contributed to the reprogramming of bacterial genetic and phenotypic identity on a planetary scale (Gillings, 2017; Gillings and Paulsen, 2014). This evolution is both tightly coupled to anthropogenic activities on the one hand and tends to promote the emergence of drug-resistant and pathogenic microbes, on the other. Further, MGEs with origins that can be traced to single bacterial strains are now observed dispersed throughout the global microbial population, a phenomenon that emerges from the interaction between microbial HGT and human infrastructures (Haraoui, 2022). Hence, although MGEs and HGT events have been critical in evolutionary flexibility, genomic innovation, and the evolution of complex life forms (Jain et al., 2003; Daubin and Szöllősi, 2016), contemporary concerns often focus on these processes as vectors for the dissemination of antimicrobial resistance genes (ARGs) and the emergence of novel pathogens (Emamalipour et al., 2020; Vos, 2020) in an era of unprecedented socio-ecological connectivity.

## 3 Contaminants of emerging concern in WWTPs and HGT events

A wide variety of emerging contaminants have been implicated in the selection and evolution of ARGs and MGEs, and their transmission. Many of these contaminants concentrate in WWTPs, including pharmaceuticals, personal care products, fungicides, biocides, and herbicides (Feng et al., 2021; Alderton et al., 2021). Disinfectants (Tan et al., 2021), heavy metals (Lin et al., 2019), preservatives (Cen et al., 2020), and organic pollutants are also implicated in the HGT of ARGs (Sharma et al., 2025) and in the evolution of metabolic genes related to pollutant metabolism and detoxification (Top and Springael, 2003; Miguel et al., 2020). Here, the focus here is on the effect of two contaminants of emerging concern on HGT and MGEs: microplastics and per- and polyfluoroalkyl substances. These contaminants are discussed due to the small but growing body of research elucidating their effects and the need for further investigation.

### 3.1 Microplastics

Microplastics (MPs), small (<5 mm) plastic particles of various polymer composition and structure, are emerging contaminants of concern in wastewater streams and aquatic, marine, estuarine and terrestrial ecosystems across the globe. MPs act as a physical substrate for microbial biofilm formation and have been demonstrated to facilitate HGT under various experimental conditions (Arias-Andres et al., 2018; Liu et al., 2022). MPs of different polymer type have been associated with increased prevalence of ARGs, MGEs, and HGT in mariculture (Liu et al., 2025) estuarine (Zhou et al., 2024), and constructed wetland (Zhao et al., 2023) environments, to name a few. This effect may be associated with the induction of oxidative stress, ROS production, and the formation of a specific “plastisphere” community that enriches MGE-harboring microorganisms (Luo et al., 2023; Liu et al., 2025).

Microplastics are increasingly shed into waste streams and produced *in situ* through the degradation of larger pieces of plastic (Zhang et al., 2021). These particles are dispersed widely in hydrological systems, bypass initial filtering in WWTPs, and may be released in effluent (Ziajahromi et al., 2016; Mason et al., 2016; Edo et al., 2020). In addition to the environments noted above, researchers have investigated the relationships between MPs and MGEs, ARGs, and HGT events in WWTP systems. For instance, Pham et al. (2021) observed that *intI1* was enriched up to 4.5-fold on microplastics incubated in a WWTP in the USA. From a functional perspective, Feng et al. (2023) demonstrated that the ARGs in MP biofilms can be horizontally transferred to free cells, demonstrating the potential correlation between gene abundance and functional effects.

Certain pollutants present in wastewater can be adsorbed onto MP surfaces resulting in localized increases in concentration and leading to bacterial stress responses, increased eDNA uptake, and lateral sharing of genetic information (Shen et al., 2023; Wang et al., 2023). For instance, Wang et al. (2021b) investigated the interactions between MPs isolated from wastewater and different pharmaceuticals. They hypothesized that the colocalization of MPs with multiple pollutants may have synergistic effects on the selection of MGEs. In agreement, their study demonstrated that microorganisms cultured with MPs adsorbed with tetracycline, triclosan, or ampicillin had significantly increased MGEs and ARGs as compared to those cultured with pharmaceuticals alone. Their findings further contribute to the model of MPs as hotspots for the selection, concentration and the transfer of MGEs. On the other hand, however, Xu M. et al.’s (2024) study suggested that MPs effectively reduced ARGs and MGEs in anaerobic digestion environments. Further, MPs were associated with a reduction in genes functionally linked to HGT which was subsequently reduced on MP surfaces. Different polymer types were linked to different effects on MGEs, with polypropylene and polyethylene having a greater effect than polyamide. The effect of MP polymeric identity on HGT and the microbial community involved (e.g., aerobic vs. anaerobic) are important areas for future research. WWTPs should continue to be a key site for these studies (Junaid et al., 2022; Syranidou and Kalogerakis, 2022).

### 3.2 Per- and polyfluoroalkyl substances

Per- and polyfluoroalkyl substances (PFAS) are a large family (>4700 in commercial use; Wang et al., 2021a) of chemically related compounds characterized by the presence of carbon-fluoride bonds and environmental and biological stability. PFAS are ecotoxic, bioaccumulative, and highly persistent in aquatic and terrestrial environments (Brunn et al., 2023). Exposure to PFAS is associated with changes to the function and structure of the human gut-associated microbiome and various environmental microbiomes (Sen et al., 2024; Beale et al., 2022; Laue et al., 2023). They are contaminants of emerging concern and are linked to health issues in humans and other species. PFAS also concentrate in WWTPs where they are removed with varying degrees of efficiency (Ilieva et al., 2024; Barisci and Suri, 2021).

A small number of studies have begun investigating the roles of PFAS substances on HGT events. For instance, Liu et al. (2023) demonstrated that low levels of two PFAS chemicals, perfluorooctanoic acid (PFOA), perfluorododecanoic acid (PFDoA) and ammonium perfluoro (2-methyl-3-oxahexanoate) (GenX) (at 0.01 and 0.1 mg/L) promoted the conjugative transfer of plasmid RP4 between *Escherichia coli*. However, higher levels (1.0 and 10 mg/L) inhibited transfer suggesting a non-linear dose response (Xu Z. et al., 2024). Mechanistic studies demonstrated that conjugative transfer was linked to oxidative stress, and higher levels of PFAS reduced ATP thereby inhibiting transfer. Another study demonstrated that PFOA can increase transmission of plasmid-encoded ARGs by up to 3.5-fold, although this was to a soil bacterial community (Yin L. et al., 2023).

Perfluorooctanoic acid also increased the risk of horizontal ARG transmission in groundwater ecosystems by selecting for denitrifying bacterial communities that harbor ARGs associated with MGEs (Chen et al., 2023). On the other hand, Chen et al. (2022) demonstrated that PFOA-induced microbial stress reduced the expression of plasmid-mediated horizontally transmissible ARGs (also in a groundwater model), demonstrating that the effects of PFAS on HGT are complex and likely depend on the microbial community involved and environmental factors. Another study demonstrates that in water distribution systems, PFAS and phthalate esters may act additively to increase MGE levels in biofilms, an effect that was also dependent upon pipe material (Yin H. et al., 2023). Furthermore, a recent study by Wang et al. (2025) demonstrated that in the presence of the quaternary ammonium compound diallyl dimethylammonium chloride, PFOA, PFHxA, and PFBS all increased the level of antimicrobial resistance genes in a nitrification community. This increase was correlated with elevated MGEs, suggesting that PFAS was involved in HGT in the community.

Collectively, these studies demonstrate that the effects of PFAS on MGEs and HGT are complex and environment dependent. A recent molecular dynamics and machine learning study by Xiao et al. (2025) predicted that PFAS increase bacterial HGT, an observation that was influenced by the electronegativity of PFAS molecules. A synergistic effect on HGT was predicted in the co-presence of PFAS and MPs. However, given the variability discussed above, it is important to validate these observations experimentally and with various microbial communities. Moreover, questions remain as to how those results obtained in model systems with controlled levels of PFAS might translate to the *in situ* activated sludge process with variable and dynamic PFAS concentrations, compositions, and distributions. Further research is also required to assess how PFAS may interact with other pollutants, stressors, and physical substrates in complex and potentially synergistic ways to affect HGT in wastewater operations.

## 4 HGT events from WWTPs associated with environmental volatility

There are complex connections between anthropogenic climate change and the selection of AMR and its transmission via MGEs and HGT. Climate change may exacerbate AMR via numerous mechanisms, including increased proximity between humans and animals (Magnano San Lio et al., 2023) altered spatial range of vectors and pathogens, and increased usage of antibiotics in humans and domesticated animals (Reverter et al., 2020). Moreover, higher temperatures are directly associated with increased rates of AMR (Meinen et al., 2023) although it has varying effects on the spread of resistance (Bagra et al., 2024a). In the Yellow river in China, for instance, ARG and MGE levels correlated with temperature (Yu et al., 2023). Other studies demonstrate that the spatial distribution (sediment or water) and mobilization potential of ARGs via MGEs differs depending on the time of year, with more MGEs in sediment during the Spring drought in Asian and European contexts (Guo et al., 2023). In the context of this review, the focus is on how climate change significantly impacts the hydrological cycle and how this influences the resistome profile of WWTPs and the spread of MGEs.

Zhou et al. (2022) identified a carbapenem-resistant *Citrobacter sedlakii* strain isolated from aerosols sampled outside a WWTP in China. Resistance was related to the presence of the blaNDM-5 gene, which was located on the IncX3 plasmid (pCSNDM-5). The plasmid could be transferred to the *Escherichia coli* recipient J53, demonstrating a possible transmission pathway for the spread of clinically relevant ABR and MGEs from WWTPs. Given that WWTP bio-aerosol generation is related to environmental conditions, including temperature, rainfall, and wind (Tian et al., 2022), Zhou et al.’s (2022) findings demonstrate the complex and interconnected pathways of MGE transmission in anthropogenic environments stemming from WWTPs. In this respect, Hou et al. (2022) investigated the effects of a severe precipitation event on the resistome of lagoon surface waters in China. Their study demonstrated that heavy rain events were associated with the promotion of HGT events by increasing the input of MGEs from diverse urban sources, such as road and agricultural runoff as well as WWTP effluent. Indeed, MGE levels were highest immediately following the storm, followed by a reduction to baseline levels that was associated with environmental controls such as pumping and floodgate opening. Urban stormwater often contains high levels of MGEs as well as selective pressures such as heavy metals, which are transported to WWTPs in sediment from roads (Zuo et al., 2022). The effects of floods and monsoons on MGE-mediated ARG dissemination is exacerbated in regions where wastewater treatment is suboptimal and untreated effluent contaminates river environments (Bagra et al., 2024b).

Collectively, these observations demonstrate the highly dynamic nature of MGE dissemination and HGT events, and highlight the temporal distribution of risk following extreme weather events. However, MGE distribution is geographically specific and dissemination depends on factors including host phylogeny (Johansson et al., 2023), suggesting that potential dissemination may be curbed by local ecology and climate. Hurricanes (Davis et al., 2020) have been associated with altered MGE profiles in watersheds, whilst a recent study has demonstrated that following a mining-associated tsunami in Brazil, MGEs were significantly increased in the disturbed river environment, among them the highly mobile and promiscuous ARG blaOXA (Suhadolnik et al., 2022). Less research has focused on the effects of droughts on the abundance of MGEs and rates of HGTs in WWTPs, but given that drought can significantly impact pollutant load in WWTPs and receiving environments (Köck-Schulmeyer et al., 2011) this is an important area for future study. Collectively, these findings demonstrate that climatic conditions are significant variables in modulating the landscape of MGEs and HGT events from WWTPs. As extreme weather events continue to increase in the modern world, their effects on the quantity, distribution, and transmission of MGEs and associated risks should become a specific focal point of research.

## 5 Transmission of genes from WWTPs to downstream organisms

The environment immediately surrounding WWTPs, and particularly the receiving waters of discharged effluent are under increased pressures. WWTPs exist in a gradient, along which the impact of anthropogenic activities can be detected, often via an increased prevalence of MGEs (Gillings et al., 2015) and ARGs. From a One Health perspective (Larsson et al., 2023), it is important to understand the connections between the MGEs discharged from WWTPs and recipient ecosystems, including environmental microbiomes, aquatic species, birds, and terrestrial organisms such as insects. In turn, these organisms can act as vectors further promoting the transmission of MGEs beyond the immediate WWTP environment.

### 5.1 Transmission to microbiomes

In the first instance, studies demonstrate that microbiomes downstream of WWTP effluent have altered community structure and dynamics (Price et al., 2018). However, it is uncertain whether these effects are related to selective chemicals acting on the receiving population, the transmission of MGEs from incoming effluent, or a combination of both. Using a nanopore approach, a study by Wu et al. (2022) demonstrated that receiving seawater contained a 10-fold enrichment of ARGs compared to clean seawater. The resistome profile matched that of the WWTP and was primarily due to the revival of microbes in the effluent that bypassed disinfection. Their results further suggested that plasmids and class I integrons were key players in the dissemination and persistence of ARGs in receiving waters. In agreement, Zhang et al. (2021) demonstrated that river microbiomes downstream of sewage treatment plants contain increased resistome diversity, due to invasion by resistance elements from WWTP effluents. These studies collectively suggest that the impacts of WWTP on HGT extend beyond the confines of the engineered ecosystem and readily infiltrate neighboring microbial communities. However, recent evidence suggests it is not only the treatment efficiency and quality of the final effluent that contributes to resistome infiltration, but also the background contamination of the river environment (Ferreira et al., 2022). This indicates that multiple stressors interact in complex ways to sculpt the mobilome of recipient ecosystems.

### 5.2 Transmission to larger organisms

The majority of studies investigating the effects of WWTP effluent on the downstream ecosystem health have involved taxonomic metagenomic studies. Such studies have demonstrated effects on the microbiomes of fish (Restivo et al., 2021), birds (Marcelino et al., 2018), mussels (Millar et al., 2022a), and insects (Millar et al., 2022b) living near WWTPs and exposed to their effluent. A comparatively smaller number of studies, however, have studied the presence and potential origins of mobile genetic elements in downstream microbial biofilms and in host microbiomes.

Insects are considered to act as vectors for the transmission of ARBs and ARGs (Gwenzi et al., 2021; Rawat et al., 2023). The feeding behavior and environmental niches of specific insects such as “filth flies” (Onwugamba et al., 2018) favors their contact with enteric pathogens potentially containing ARGs. In terms of HGT, events Doud et al. (2014) observed that house flies (*Musca domestica*) isolated from WWTPs and neighboring urban areas contain *Enterococcus faecalis* clones resistant to several antibiotics. Resistance was horizontally transferable between species as demonstrated by *in vitro* conjugation experiments. Genetic experiments (PFGE) suggested that the isolates were from an agricultural source, but higher resolution sequencing studies would be necessary to investigate this hypothesis. Future experiments should continue to use high resolution metagenomic sequencing to trace the origins of AMR genes in insect species living near WWTPs. On the other hand, Lee et al. (2023) demonstrated that WWTP-originating ARGs were not enriched in amphipods in receiving environments, thereby demonstrating the species and ecosystem-dependence of HGT in the vicinity of WWTPs.

In terms of larger organisms, the microbiomes of *Hemiculter leucisculus* living downstream of WWTPs demonstrated increased co-occurence of MGEs and ARGs (Xue et al., 2021), suggesting that the MGEs from WWTP effluent infiltrated fish microbiota (Guan et al., 2022). Specifically, the findings suggested that plasmids were key vectors for the transmission of ARGs along an anthropogenic gradient from WWTP to pristine river environments. Gulls are considered a key organism involved in the wide-range transmission of ARGs between different species due to their intimate connections across human and aquatic environments (Wyrsch et al., 2022; Lin et al., 2020; Zeballos-Gross et al., 2021; Marcelino et al., 2018). Indeed, gulls living in proximity to anthropogenic environments hosted *Escherichia coli* and *Klebsiella pneumoniae* strains resistant to clinically important antibiotics, with some determinants carried on plasmids (Mukerji et al., 2023). Further, Woksepp et al. (2023) detected carbapenemase-producing *Enterobacterales* (CPE) in Swedish wastewater and gull feces. Four different carbapenemases were identified (blaGES-5, blaIMI-3, blaOXA-181 and blaOXA-244) which were carried on plasmids [IncP and IncFII(Yp)]. Those MGEs detected in the gull feces were strongly suggested to have been acquired from the WWTP where the authors had observed the birds feeding. These results are concerning, especially given that certain carbapenemases and their MGEs were detected in gull feces 2 km from the WWTP.

## 6 Conclusion

This minireview has focused on three scales and domains of HGT in and around WWTPs that are emerging in the scientific literature. However, it is important to consider the inseparability and connectivity between these processes. Researchers should continue to analyze the role of specific factors, such as micropollutants, in the dynamics of HGT but more integrated perspectives are also necessary, such as “One Health,” “Global Health” or “Planetary Health” approaches (Horvat and Kovačević, 2025; Ramsamy et al., 2022). WWTPs are themselves sites of integration for municipal, industrial, and agricultural effluent, microbial evolution, and increasingly volatile climate regimes. On the one hand, the studies summarized here point to general trends in which WWTPs act as catalysts for HGT and the selective accumulation of MGEs. On the other hand, the growing research also points to important differences that are site, context, and microbial community-dependent. These elements of variability underscore the need to understand bacterial dynamics on a case-by-case basis, even as their effects reverberate through larger systems. Thus, whilst an interconnected picture begins to link WWTPs and HGT with novel and emerging contaminants, volatile weather, and trophic links to wider ecosystems, future research is necessary to explore these connections across specific environments and socio-ecological systems.
